# Burden of hepatitis E virus infection in pregnancy and maternofoetal outcomes: a systematic review and meta-analysis

**DOI:** 10.1186/s12884-020-03116-2

**Published:** 2020-07-28

**Authors:** Jean Joel Bigna, Abdou Fatawou Modiyinji, Jobert Richie Nansseu, Marie A. Amougou, Moise Nola, Sébastien Kenmoe, Elvis Temfack, Richard Njouom

**Affiliations:** 1 Department of Epidemiology and Public Health, Centre Pasteur of Cameroon, P.O. Box 1274, Yaoundé, Cameroon; 2 Department of Virology, Centre Pasteur of Cameroon, Yaoundé, Cameroon; 3 Department of Animals Biology and Physiology, Faculty of Sciences, University of Yaoundé I, Yaoundé, Cameroon; 4 Department for the Control of Disease, Epidemics and Pandemics, Ministry of Public Health, Yaoundé, Cameroon; 5 Department of Public Health, Faculty of Medicine and Biomedical Sciences, University of Yaoundé I, Yaoundé, Cameroon; 6 Department of Biochemistry, Faculty of Sciences, University of Yaoundé I, Yaoundé, Cameroon; 7 Department of Internal Medicine, Douala General Hospital, Douala, Cameroon

**Keywords:** Hepatitis E, Pregnancy, Women health, Vertical transmission, Maternal deaths, Intrauterine deaths, Miscarriage, Low birth weight, Preterm, Small for gestational age

## Abstract

**Background:**

There is still a dearth of knowledge on the burden of HEV infection in the global population of pregnant women. Therefore, we conducted a systematic review and meta-analysis to estimate the global burden of HEV infection in pregnancy.

**Methods:**

We searched PubMed, Embase, Web of Knowledge, and Global Index Medicus to identify articles published until January 26, 2020. We considered cross-sectional, case-control, and cohort studies reporting the immunoglobulins M HEV seroprevalence in asymptomatic and symptomatic (jaundice or elevated transaminases) pregnant women or investigating the association between HEV infection and maternofoetal outcomes. We used a random-effects model to pool studies. This review was registered with PROSPERO, CRD42018093820.

**Results:**

For HEV prevalence estimates, we included 52 studies (11,663 pregnant women). The seroprevalence was 3.5% (95% confidence interval: 1.4–6.4) in asymptomatic women (most of whom from high endemic areas). The prevalence in symptomatic women was 49.6% (42.6–56.7) with data only from HEV high endemic countries. In the multivariable meta-regression model, the prevalence was higher in symptomatic women compared to asymptomatic (adjusted prevalence odds ratio [aPOR]: 1.76; 95%CI: 1.61–1.91) and decreased with increasing year of publication (by 10-year) (aPOR: 0.90; 95%CI: 0.84–0.96). The proportion of HEV vertical transmission was 36.9% (13.3–64.2). Risk of bias was low, moderate and high respectively in 12 (23%), 37 (70%), and 4 studies (7%) addressing HEV prevalence estimation. HEV infection was associated with maternal deaths (pooled OR 7.17; 3.32–15.47), low birth weight (OR: 3.23; 1.71–6.10), small for gestational age (OR: 3.63; 1.25–10.49), preterm < 32 weeks (OR: 4.18; 1.23–14.20), and preterm < 37 weeks (OR: 3.45; 2.32–5.13), stillbirth (OR: 2.61; 1.64–4.14), intrauterine deaths (OR: 3.07; 2.13–4.43), and not with miscarriage (OR: 1.74; 0.77–3.90). All studies which assessed the association between HEV infection and maternofoetal outcomes had a moderate risk of bias.

**Conclusions:**

Findings from this study are suggestive of a high burden of HEV infection in pregnancy in high endemic countries, its association with poor maternofoetal outcomes, and a high rate of vertical transmission. This study supports the need for specific strategies to prevent exposure of pregnant women to HEV infection, especially in high endemic areas.

## Background

In 2016, the World Health Organization (WHO) launched a global strategy to halt the transmission of viral hepatitis supporting that people living with viral hepatitis should have access to safe, affordable, and effective prevention, care and treatment services [1]. Specifically, the aims by 2030 are to reduce by 90% the number of new cases of hepatitis, to treat 80% of eligible people infected with viral hepatitis so as to reduce by 65% the number of hepatitis related deaths [1]. Globally, it was estimated that about 1.34 million deaths which occurred in 2015 were due to viral hepatitis, of whom 95% were attributable to hepatitis B and C chronic infections, and those remaining, to hepatitis A and E infections [1, 2]. For the specific case of hepatitis E infection, global estimates indicate that about 20 million new cases of hepatitis E virus (HEV) infections occur each year, 3.3 million of whom are symptomatic [3]. In 2015, WHO reported approximately 44,000 fatal cases of HEV, accounting for about 3.3% of all viral hepatitis related mortality [3].

HEV is a water- and food-borne infection that can potentially cause acute outbreaks in populations with poor sanitation [1, 3]. However, zoonotic and transfusion-related transmission have also been documented [4, 5]. To date, no specific treatment exists for HEV infection; as a consequence, its management relies mostly on supportive care [1, 3]. On the other hand, prevention is oriented towards reducing exposure by improved sanitation, safe food and drinking, and vaccination [1]. Compared to hepatitis B and C, HEV infection is more unlikely to result in chronic liver disease and progression to fulminant hepatitis though rare, is mostly driven by host-specific than virus-specific factors [6]. Nevertheless, fulminant hepatitis occurs more frequently during pregnancy [3].

Mechanisms for fulminant hepatitis during pregnancy include lower CD_4_/CD_8_ cells ratio and increased levels of steroid hormones [7], reduced progesterone receptor expression, higher interleukin and viral load [8, 9]. Consequently, pregnant women with HEV, particularly those in the second and third trimester, are at higher risk of poor maternofoetal outcomes as suggested by narrative reviews of observational studies [3, 9, 10]. However and to the very best of our knowledge, there remains a dearth of knowledge on the burden of HEV infection among pregnant women living in high endemic countries. Therefore, this systematic review and meta-analysis was conducted to estimate the prevalence of HEV in pregnancy as well as its association with maternofoetal outcomes.

## Methods

This systematic review was registered in the PROSPERO International Prospective Register of systematic reviews, registration number CRD42018093820. We used the Preferred Reporting Items for Systematic Reviews and Meta-Analyses guidelines to report this review [11].

### Search strategy and selection criteria

We carried-out a comprehensive search on major electronic databases including MEDLINE (through PubMed), EMBASE, Web of Knowledge, and Global Index Medicus to identify relevant studies on HEV infection among pregnant women. The search strategy was adapted to suit each database as illustrated by the search on PubMed (Supplementary Table 1). We considered studies published until January 26, 2020, without any language or country restriction. To supplement the electronic search, references of all relevant studies were also screened for potential consideration.

Two review authors (AF and SK) independently screened titles and abstracts of aggregated citations retrieved from the electronic search, and full texts of potentially eligible articles were further assessed for inclusion. Disagreements were resolved through discussion and unreached consensus was resolved by a third author (JJB).

Cross-sectional, case-control, and cohort studies were considered for inclusion. We excluded letters, reviews, commentaries, editorials, and studies without primary data. We also excluded studies that included participants who had been selected based on presence of other viral hepatitis or HIV and the description of method was incomplete. In these considered studies, HEV infection had to be diagnosed by serum detection of immunoglobulins (Ig) the major outcomes of interest, comparing HEV positive and negative pregnant women, included maternal mortality, foetal immaturity (low birth weight, preterm birth, small for gestational age), and foetal non-survival (intrauterine death, miscarriage, stillbirth). To estimate the prevalence of HEV vertical transmission, we calculated the proportion of HEV infected new-borns (HEV positive with polymerase chain reaction technique on neonatal cord-blood or peripheral blood from the new-borns) among HEV infected mothers.

### Data extraction and management

Using a pretested data extraction form, two review authors (JJB and AFM) independently extracted relevant information, including first author, publication year and period of participants’ recruitment, country, site, area, setting, timing of data collection, study design, sampling method, sample size, sample tested for HEV, number of participants with IgM of HEV in blood or stool, number of participants with maternofoetal complications and the WHO region. Additionally, for each country of study recruitment, we retrieved data on human development index (HDI) [12]. We defined two groups of pregnant women based on clinical presentation at HEV screening: symptomatic and asymptomatic pregnant women. Symptomatic women were those with signs suggestive of acute hepatitis including jaundice and/or elevated transaminases. When relevant data from included studies were not available, corresponding authors were contacted at least twice for clarification. For the methodological quality and risk of bias assessment of included studies, the tool to be used was determined by the outcome of interest which guided study inclusion. Accordingly, for studies presenting the prevalence of HEV, we used an adapted version of the tool developed by Hoy and colleagues (Supplementary Table 2) [13]; for those presenting the association between HEV infection and maternofoetal outcomes, we used an adapted version of the ROBINS-I tool (Supplementary Table 3) [14]. Two review authors (AFM and JJB) independently ran the assessment; discrepancies were arbitrated by a third review author (JRN). Inter-rater agreements between investigators for study inclusion and methodological quality assessment were assessed using the Cohen’s κ coefficient [15].

### Data synthesis and analysis

We undertook data meta-analysis using the package ‘*meta*’ (version 4.9–2) of R (version 3.6.2, The R Foundation for Statistical Computing). For HEV seroprevalence estimates, we calculated unadjusted prevalence based on crude numerators and denominators provided by individual studies. Then, to minimise the effect of the size of study-specific estimates of prevalence on the overall estimate, we used the Freeman–Tukey double arc-sine transformation before pooling data with a random-effects meta-analysis [16]. We also performed sensitivity analyses to assess the robustness of our estimates when only studies with a low risk of bias were included. Symmetry of counter-enhanced funnel plots and the Egger test were used to assess reporting and publication bias [17]. Consideration of significant publication bias was at a threshold of a *p*-value < 0.10. To assess the association between HEV infection and maternofoetal outcomes, we used random-effects approach by the Der Simonian and Laird method, and reported pooled weighted results as odds ratios (OR) both with 95% confidence and 95% prediction intervals (CI and PI) [18]. A continuous correction of 0.5 was added to each cell frequency for studies with a zero cell count. Heterogeneity across studies was assessed by χ^2^ test, and reported as I^2^ statistics [19]. In the case of substantial heterogeneity (I^2^ > 50%) [20], we carried-out subgroup analysis to investigate sources of residual heterogeneity.

Univariable meta-regression analysis was performed to identify and quantify (R^2^) sources of heterogeneity including clinical presentation, year of publication, HDI, WHO regions, country HEV endemic profile, and sampling method. We planned to integrate clinical profile in the final multivariable meta-regression model that was chosen based on the lowest corrected Akaike’s information criterion (AICc). A *p* value < 0.05 was considered statistically significant. Strength of association were reported with (adjusted) prevalence odds ratios (aPOR) and corresponding 95% CIs.

## Results

### Study selection and characteristics

In total, we identified 597 records, of which 54 were finally included [8, 21–73] (Supplementary Fig. 1). Agreement between review authors for study selection based on title and abstract (κ = 0.89) and data extraction (κ = 0.78) were moderate to high. Among the 54 included studies performed in 22 countries, 51 had been conducted to estimate HEV prevalence (Supplementary Table 4) and 5 to investigate the association between HEV and maternofoetal outcomes (Supplementary Table 5). Risk of bias was low, moderate and high respectively in 12 (23%), 37 (70%), and 4 studies (7%) addressing HEV prevalence estimation; all studies which assessed the association between HEV infection and maternofoetal outcomes had a moderate risk of bias. Overall, 53 (98%) studies were cross-sectional and one (2%) was case control. In the countries where studies were done, 29 (54%) were in South-East Asia, 10 in Eastern Mediterranean (18%), 6 in Africa (11%), 4 (7%) in Europe, 3 (6%) in Western Pacific, and 2 (4%) in The Americas. Clinically, pregnant women were symptomatic in 29 studies (54%) (Supplementary Table 6). The prevalence of viral hepatitis A, viral hepatitis B, viral hepatitis C, and viral hepatitis D varied from 0 to 14.6% (*n* = 23 studies), from 0 to 31.3% (*n* = 27 studies), from 0 to 13.5% (*n* = 23 studies), and from 0 to 1.5% (*n* = 3 studies), respectively.

### Global prevalence of HEV infection in pregnancy and vertical transmission

**Fig. 1:**
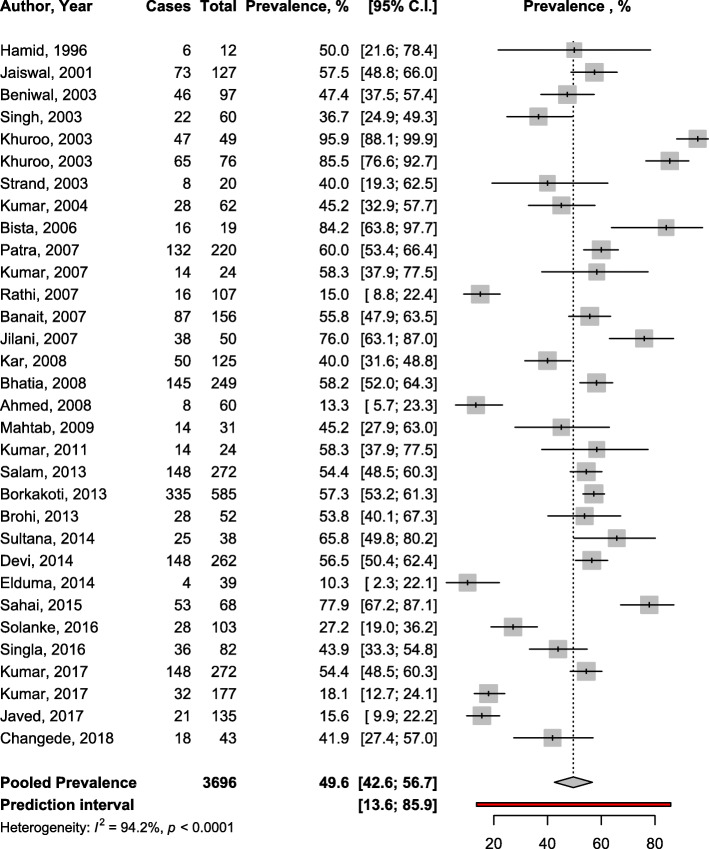
Forest plot of the meta-analysis of IgM seroprevalence of HEV infection among symptomatic pregnant women

**Fig. 2:**
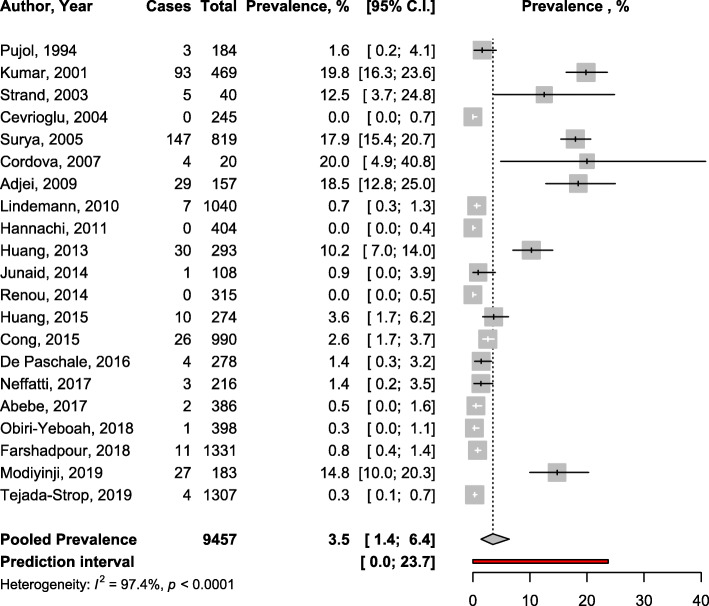
Forest plot of the meta-analysis of IgM seroprevalence of HEV infection among asymptomatic pregnant women

To estimate the prevalence of HEV, a total of 13,153 pregnant women were included in the meta-analysis. All data on symptomatic women were only from high HEV endemic countries. In asymptomatic women, 14 studies were from high endemic, 3 from endemic, and 4 from not endemic countries. The HEV infection prevalence was 49.6% (95%CI: 42.6–56.7) in symptomatic (Fig. 1) and 3.5% (95%CI: 1.4–6.4) in asymptomatic pregnant women (Fig. 2) with substantial heterogeneity; *p* <  0.0001 (Table 1). Funnel plots suggested no asymmetry (Supplementary Figs. 2 and 3) confirmed by the Egger test (Table 1).

**Table 1:** Meta-analysis prevalence of hepatitis E virus infection in the global population of pregnant women

	Prevalence (95% confidence intervals)	95% prediction intervals	*N* Studies	*N* Participants	Heterogeneity			*p* Egger test	*p* difference
					H (95% confidence intervals)	I^2^ (95% confidence intervals)	*p*		
Symptomatic	49.6 (42.6–56.7)	13.6–85.9	32	3696	4.2 (3.7–4.6)	94.2 (92.7–95.4)	< 0.0001	0.739	< 0.0001
Asymptomatic	3.4 (1.2–6.4)	0.0–24.3	19	7967	6.1 (5.4–6.8)	97.3 (96.6–97.9)	< 0.0001	0.633	
***By HDI***									
Asymptomatic									
Low and medium HDI	5.0 (0.8–11.9)	0.0–41.6	9	3678	7.3 (6.2–8.5)	98.1 (97.4–98.6)	< 0.0001	0.551	0.410
High and very high HDI	2.6 (0.6–5.5)	0.0–19.6	12	5781	5.4 (4.6–6.4)	96.6 (95.3–97.5)	< 0.0001	0.473	
Symptomatic									
Low and medium HDI	49.6 (42.6–56.7)	13.6–85.9	32	3696	4.2 (3.7–4.6)	94.2 (92.7–95.4)	< 0.0001	0.739	NA
High and very high HDI	–	–	0	–	–	–	–	–	

*HDI* human development index

There was no difference in HEV prevalence considering HDI grouping for asymptomatic women (Table 1). There was no data on symptomatic women from high HDI countries (Table 1).

In the univariable meta-regression analysis, the HEV prevalence was associated with clinical presentation (R^2^: 76.1%), year of publication (R^2^; 9.8%), human development index (R^2^: 24.6%), WHO regions (65.3%), and HEV Endemic profile of countries (R^2^: 0.0%) (Table 2). In the final multivariable model, two variables were included: clinical profile and year of publication explaining 80.6% of the variance of HEV prevalence. The prevalence was higher in symptomatic women compared to asymptomatic (aPOR: 1.76; 95%CI: 1.61–1.91; *p* <  0.0001) and decreased with increasing year of publication (by 10-year) (aPOR: 0.90; 95%CI: 0.84–0.96; *p* = 0.003) (Table 2).

**Table 2:** Meta-regression analysis of HEV infection prevalence in global population of pregnant women

Variables (reference)	Univariable model			Explained variance, R^**2**^	Multivariable model	
	Prevalence odds ratio (95% confidence intervals)	***P*** value	***P*** value, test for moderator		Adjusted prevalence odds ratio (95% confidence intervals)	***P*** value
**Clinical presentation (asymptomatic)**			< 0.0001	76.1%		
Symptomatic	1.79 (1.64–1.97)	< 0.0001			1.76 (1.61–1.91)	< 0.0001
**Year of publication**			0.012	9.8%		
By increase of 10 years	0.84 (0.73–0.96)	0.012			0.90 (0.84–0.96)	0.003
**Human development index (high and very high)**			< 0.0001	24.6%		
Low and medium	1.62 (1.35–1.95)	< 0.0001				
**Regions (Africa)**			< 0.0001	65.3%		
Americas	0.93 (0.71–1.22)	0.615				
Eastern Mediterranean	1.16 (0.96–1.39)	0.132				
Europe	0.80 (0.62–1.04)	0.092				
South-East Asia	1.71 (1.46–2.00)	< 0.0001				
Western Pacific	0.96 (0.74–1.24)	0.744				
**Country HEV endemic profile (low)**			0.031	0.0%		
Endemic	0.91 (0.55–1.50)	0.717				
High	1.37 (0.98–1.92)	0.069				
**Sampling (non-probability-based)**			0.734	0.0%		
Probability-based	1.11 (0.68–1.79)	0.682				
Unclear	1.07 (0.89–1.29)	0.460				

**Fig. 3:**
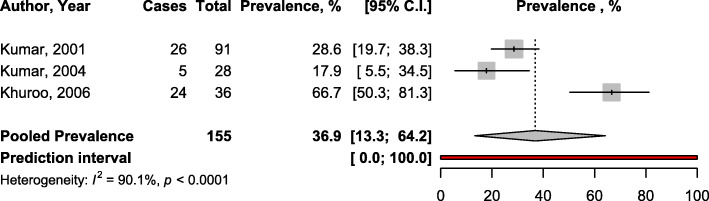
Forest plot of the meta-analysis prevalence of vertical transmission

Three studies with a total of 155 women reported data on HEV vertical transmission. The pooled estimate of vertical transmission was 36.9% (95%CI 13.3–64.2) (Fig. 3). All three studies diagnosed HEV infection using neonatal cord-blood samples.

### Maternofoetal outcomes in HEV infection

**Fig. 4:**
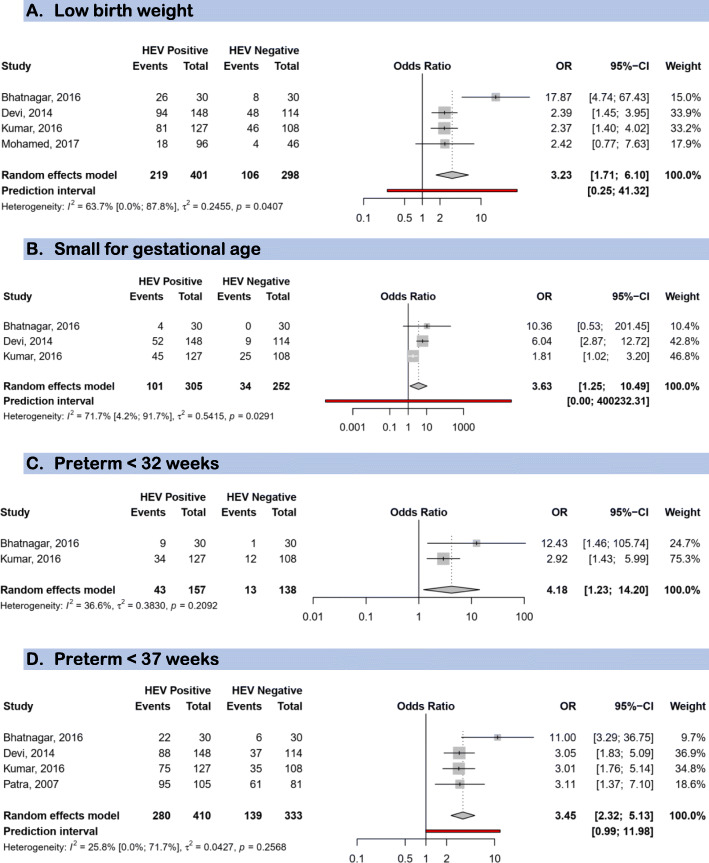
Forest plot of the risk of foetal immaturity associated with HEV infection during pregnancy. **a** Low birth weight. **b** Small for gestational age. **c** Preterm < 32 weeks. **d** Preterm < 37 weeks

**Fig. 5:**
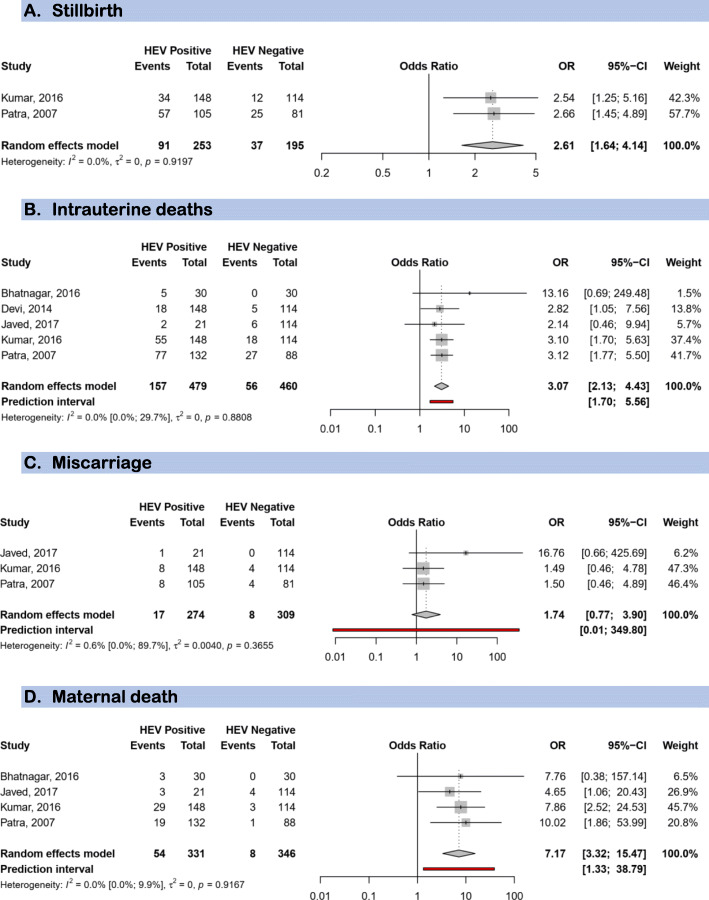
Forest plot of the risk of foetal and maternal mortality associated with HEV infection during pregnancy. **a** Stillbirth. **b** Intrauterine deaths. **c** Miscarriage. **d** Maternal death

In total, 479 HEV positive and 490 HEV negative women from five studies were included to investigate the association between HEV infection and maternofoetal outcomes. HEV infection during pregnancy was associated with low birth weight (OR: 3.23; 95%CI: 1.71–6.10), small for gestational age (OR: 3.63; 95%CI: 1.25–10.49), preterm < 32 weeks (OR: 4.18; 95%CI: 1.23–14.20), and preterm < 37 weeks (OR: 3.45; 95%CI: 2.32–5.13) (Fig. 4). HEV infection during pregnancy was also associated with stillbirth (OR: 2.61; 95%CI: 1.64–4.14), intrauterine deaths (OR: 3.07; 95%CI: 2.13–4.43), but not with miscarriage (OR: 1.74; 95%CI: 0.77–3.90) (Fig. 5). HEV infection during pregnancy increased the likelihood of maternal deaths (pooled OR 7.17, 95%CI 3.32–15.47) (Fig. 5).

## Discussion

To the best of our knowledge, this is the first systematic review and meta-analysis which estimated the burden of HEV infection in pregnancy. Our main findings show a high burden of HEV infection in pregnant women, especially among symptomatic women. In addition, HEV in pregnancy was associated with a two- to three-fold increase in intrauterine foetal demise, a three-fold increase in poor intrauterine foetal maturity, and a significant increase in the likelihood of maternal death. What’s more, HEV prevalence decreased overtime among pregnant women.

Previous modelling studies through a Global Burden of Disease approach [74] estimated the HEV seroprevalence in the general population aged 15 to 45 (childbearing age for women) years to be between 5 and 22%. Our finding among pregnant women is concordant with those estimates. Available evidence shows that apart from non-infectious causes, hepatitis E is a significant cause of jaundice in pregnancy because pregnant women are more vulnerable to HEV infection than to other viral hepatitis (A, B, and C) [75]. The high IgM seroprevalence (almost 50%) of HEV infection in symptomatic women may be explained by the fact that all studies included in this analysis were from HEV endemic areas. We found that higher HEV infection prevalence was associated with low HDI, however without a significant difference. In fact, HEV infection is a disease of resource-limited settings with poor sanitation and hygiene services leading to water and food contaminations [3]. Indeed, HEV infection is known as a disease of financial, educational, and infrastructural poverty [3]. Although the current analyses pleaded for a decreasing trends of HEV prevalence overtime, specific attention to curb the burden of HEV infection is needed for high HEV endemic areas. However, this finding should be interpreted with caution since incidence data are better to estimate the dynamic of an infection, rather than prevalence data.

Narrative reviews suggested that HEV infection during pregnancy is a risk factor for poor maternal and foetal outcomes, in particular at a later stage of pregnancy [3, 9, 10]. Although the physiopathology of HEV infection in pregnancy and its outcomes is not yet clear enough, it is possible that there exists an interplay between hormonal (reduced oestrogen and progesterone receptor expression) and immunologic changes (maintenance of the antigenic foetus in the maternal environment by suppression of T cell mediated immunity) during pregnancy, alongside a HEV high viral load [8, 76]. Physiological changes in hormonal and immunologic interplay which should normally favour pregnancy evolution, become deleterious to both the foetus and the mother, affecting all stages of foetal growth and maturity. For foetal non-survival, only miscarriage was not associated with HEV infection. At the debut of the pregnancy, the interplay between changes during pregnancy and presence of HEV may not be sufficiently important to increase significantly the risk of miscarriage. During the first 20 weeks of pregnancy, the plasma concentration of cytokines begins to decrease [9]. This gradual decrease in the level of cytokines leads to a low concentration of the plasma levels of cytokines in late pregnancy, hence giving rise to a decrease in immunity. This is why HEV infection may be associated with a higher risk of poor foetal outcomes in late pregnancy [9].

We found that the odds of death during pregnancy increased by 7 times in the presence of HEV infection. During pregnancy, there is a high production of steroid hormones [8, 9]. These steroid hormones may promote viral replication. The interplay between immunological and hormonal factors and between the virus and the host leads to severe liver damage in pregnancy, increasing the risk for fulminant hepatic failure and subsequently, that for death.

To date, there is no definitive curative treatment for HEV infection; therefore, strategies for curbing its burden should be focussed on prevention. Actually, only one licensed hepatitis E vaccine is considered promising, which is licensed only in China [77]. There is no actual evidence to recommend the use of any hepatitis vaccine systematically through national programs including pregnant women [77]. The vaccine is recommended for people with a high risk of HEV infection and could therefore be used among pregnant women living in endemic areas or where HEV outbreaks are more likely to occur [3]. While waiting for further developments of hepatitis E vaccines, since HEV infection is a food-borne infection, health policy makers and stakeholders should focus on strategies to improve hygiene, sanitation, water supplies and food safety that can be integrated in a global development program. Indeed, HEV is a disease of poverty [3]. More research is needed to better understand how HEV impacts negatively the course of pregnancy. Research is also warranted to develop innovative community-based strategies to curb the burden of food-borne diseases including HEV infection and to develop antiviral treatments against acute HEV infection.

Nevertheless, findings from this study should be interpreted with caution. Actually, the review was limited by the scarcity of studies reporting some of the outcomes of interest. There were not enough studies to conduct meta-regression and sub-group analysis in order to identify modifiers of the association between HEV infection and poor pregnancy outcomes. In the estimation of the global prevalence, not all countries were represented from all WHO regions, which may limit the generalizability of current estimates. This also limited the investigation of sources of heterogeneity of HEV prevalence by WHO regions. It was difficult to assess all assays used and the prevalence of other viral hepatitis as sources of heterogeneity since they were inconsistently reported and not reported in most of original studies.

Notwithstanding, this first systematic review with meta-analyses summarised data on the prevalence of HEV infection in pregnant women and on the association between maternal and foetal outcomes in pregnancy. Another strength of this study relies in the absence of any publication bias. In addition, the between-studies heterogeneity was not significant when investigating association between HEV infection and pregnancy outcomes.

## Conclusions

The present findings suggest a high burden of HEV infection in pregnancy in high endemic countries, its association with poor maternal and foetal outcomes, and a high rate of vertical transmission. As such, this study supports the need for specific strategies to prevent exposure of pregnant women to HEV infection, especially in resource-limited settings, areas where HEV outbreaks are more likely to occur, and in HEV high endemic areas. The widespread use of hepatitis E vaccine among pregnant women in these settings and areas should be explored. Moreover, further studies are needed to identify plausible causal pathways for a better description of the association between HEV infection and poor maternal and foetal outcomes.

## Supplementary information

**Additional file 1: Table S1.** Search strategy in PubMed. **Table S2.** Risk of bias tool. **Table S3.** Risk of bias on study investigating the association between HEV infection and maternofetal outcomes. **Table S4.** Individual characteristics of studies included in the meta-analysis prevalence of HEV infection in the global population of pregnant women. **Table S5.** Individual characteristics of studies included in the meta-analysis of pregnancy outcomes in HEV infection in the global population of pregnant women. **Table S6.** Characteristics of studies included in the meta-analysis. **Figure S1.** The review process. **Figure S2.** Funnel plot for HEV prevalence in asymptomatic pregnant women. **Figure S3.** Funnel plot for HEV prevalence in symptomatic pregnant women.

## Data Availability

All data generated or analyzed during this study are included in this published article and its supplementary information files.

## Abbreviations

HEV: Hepatitis E
WHO: World Health Organization
