# Supplementary material for: Burden of hepatitis E virus infection in pregnancy and maternofoetal outcomes: a systematic review and meta-analysis
Source: BMC Pregnancy Childbirth. 2020 Jul 28;20:426. doi: 10.1186/s12884-020-03116-2 (PMC7388479; doi:10.1186/s12884-020-03116-2)
Supplement: Supplementary file 1 — Additional file 1: Table S1. Search strategy in PubMed. Table S2. Risk of bias tool. Table S3. Risk of bias on study investigating the association between HEV infection and maternofetal outcomes. Table S4. Individual characteristics of studies included in the meta-analysis prevalence of HEV infection in the global population of pregnant women. Table S5. Individual characteristics of studies included in the meta-analysis of pregnancy outcomes in HEV infection in the global population of pregnant women. Table S6. Characteristics of studies included in the meta-analysis. Figure S1. The review process. Figure S2. Funnel plot for HEV prevalence in asymptomatic pregnant women. Figure S3. Funnel plot for HEV prevalence in symptomatic pregnant women. [file 12884_2020_3116_MOESM1_ESM.pdf]

Burden of hepatitis E virus infection in pregnancy and maternofoetal outcomes: a systematic review and meta-analysis

Jean Joel Bigna, MD, MPH; Abdou Fatawou Modiyinji, MSc; Jobert Richie Nansseu, MD; Marie A. Amougou, MSc; Moise Nola, MSc, PhD; Sébastien Kenmoe, MSc, PhD; Elvis Temfack, MD, MSc, PhD; Richard Njouom, MSc, PhD

APPENDIX

Supplementary Table 1. Search strategy in PubMed 2
Supplementary Table 2 : Risk of bias tool 3
Supplementary Table 3 : Risk of bias on study investigating the association between HEV infection and maternofetal outcomes 5
Supplementary Table 4 : Individual characteristics of studies included in the meta-analysis prevalence of HEV infection in the global population of pregnant women 6
Supplementary Table 5 : Individual characteristics of studies included in the meta-analysis of pregnancy outcomes in HEV infection in the global population of pregnant women 9
Supplementary Table 6 : Characteristics of studies included in the meta-analysis 10

Supplementary Figure 1 : The review process 10
Supplementary Figure 2 : Funnel plot for HEV prevalence in asymptomatic pregnant women 12
Supplementary Figure 3 : Funnel plot for HEV prevalence in symptomatic pregnant women 12

Supplementary Table 1. ***Search strategy in PubMed***

**Search**	**Search terms**
#1	"hepatitis E" OR "viral hepatitis E" OR "hepatitis E virus" OR "hepatitis E antibody" OR "hepatitis E antigen"
#2	"hepatitis non A non B" OR "hepatitis non A non B non C" OR "hepatitis virus non A non B"
#3	#1 OR #2
#4	"pregnancy" OR "pregnant women" OR "antenatal" OR pregnan* OR "prenatal" OR gravidity OR parturition OR maternal
#5	Fetal OR foetal OR fetomaternal OR maternofetal OR maternofoetal OR foeto-maternal OR fetus
#6	#4 OR #5
#7	#3 AND #6

Supplementary Table 2 ***: Risk of bias tool***

**Risk of Bias Item**	**Yes (Low Risk) or No (High risk)**
**External Validity**	
1. Was the study target population a close representation of the pregnant population in relation to relevant variables?	
2. Was the sampling frame a true or close representation of the target population?	
3. Was some form of random selection used to select the sample, OR, was a census undertaken?	
4. Was the likelihood of non-participation bias minimal?	
**Internal Validity**	
5. Were data collected directly from the subjects (as opposed to medical records)?	
6. Were acceptable case definition of HEV used?	
7. Was a reliable and accepted diagnosis method for HEV utilised?	
8. Was the same mode of data collection used for all subjects?	
9. Was the length of the shortest prevalence period for the parameter of interest appropriate?	
10. Were the numerator(s) and denominator(s) for the calculation of the prevalence of dyslipidemia appropriate?	
11. Summary item on the overall risk of study bias<br><br>LOW RISK OF BIAS: 8 or more "yes" answers. Further research is very unlikely to change our confidence in the estimate.<br><br>MODERATE RISK OF BIAS: 6 to 7 "yes" answers. Further research is likely to have an important impact on our confidence in the estimate and may change the estimate.	

HIGH RISK OF BIAS: 5 or fewer “yes” answers. Further research is very likely to have an important impact on our confidence in the estimate and is likely to change the estimate.	

Supplementary Table 3 : ***Risk of bias on study investigating the association between HEV infection and maternofetal outcomes***

Author	Year	Is there potential for confounding of the effect of HEV in this study?	Did the authors use an appropriate analysis method that controlled for all the important confounding domains?	Bias due to confounding	Was selection of participants into the study (or into the analysis) different between women with HEV and women without HEV?	Bias in selection	Were exposure/non exposure groups clearly defined?	Could classification of HEV exposure or not exposure status have been affected by knowledge of the outcome or risk of the outcome?	Bias in classification of exposure	Were outcome data available for all, or nearly all, participants?	Were participants excluded due to missing data on other variables needed for the analysis?	Bias due to missing data	Could the outcome measure have been influenced by knowledge of the exposure received?	Were outcome assessors aware of the HEV exposure by study participants?	Were the methods of outcome assessment comparable across HEV positive and HEV negative groups?	Bias in Outcome Measurement	Is the reported effect estimate likely to be selected, on the basis of the results, from multiple outcome measurements within the outcome domain?	Is the reported effect estimate likely to be selected, on the basis of the results, from multiple analyses of the exposure-outcome relationship?	Is the reported effect estimate likely to be selected, on the basis of the results, from different subgroups?	Bias in selection of report domain	**Global**
Kumar	2014	Probably No	No	Moderate	No	Low	Yes	No	Low	Yes	No	Low	No	No Information	Yes	Moderate	No	No	No	Low	**Moderate**
Devi	2014	Probably No	No	Moderate	No	Low	Yes	No	Low	Yes	No	Low	No	No Information	Yes	Moderate	No	No	No	Low	**Moderate**
Bhatnagar	2016	Probably No	No	Moderate	No	Low	Yes	No	Low	Yes	No	Low	No	No Information	Yes	Moderate	No	No	No	Low	**Moderate**
Javed	2017	Probably No	No	Moderate	No	Low	Yes	No	Low	Yes	No	Low	No	No Information	Yes	Moderate	No	No	No	Low	**Moderate**
Patra	2007	Probably No	No	Moderate	No	Low	Yes	No	Low	Yes	No	Low	No	No Information	Yes	Moderate	No	No	No	Low	**Moderate**

Supplementary Table 4 : ***Individual characteristics of studies included in the meta-analysis prevalence of HEV infection in the global population of pregnant women***

Author	Year	Sample	Clinical presentation	Risk of bias	Country	Period	Design	Number of centers	Area	Setting	Timing	Sampling	Age, y	%HAV	%HBV	%HCV	%HDV	%Nulliparous
Abebe	**2017**	386	None	Low	Ethiopia	2014-2015	Cross-sectional	Multi-center	Urban	Other hospital based (not antenatal care)	Prospective	Consecutive	28.9	NR	NR	NR	NR	NR
Adjei	**2009**	157	None	Moderate	Ghana	2008	Cross-sectional	Single-center	Urban	Other hospital based (not antenatal care)	Prospective	Consecutive	28.89	NR	NR	NR	NR	NR
Ahmed	**2008**	60	AVH	Moderate	Sudan	2007	Cross-sectional	Multi-center	Urban	Other hospital based (not antenatal care)	Prospective	Consecutive	26.8	NR	31.30%	6.30%	NR	NR
Banait	**2007**	156	Liver dysfunction	Moderate	India	2003-2004	Cross-sectional	Multi-center	Urban	Other hospital based (not antenatal care)	Retrospective	Unclear/not described	25.5	NR	1.30%	NR	NR	28.6
Beniwal	**2003**	97	Liver dysfunction	Moderate	India	2000-2002	Cross-sectional	Single-center	Urban	Other hospital based (not antenatal care)	Prospective	Consecutive	NR	5.20%	7.20%	0	NR	0
Bhatia	**2008**	249	AVH	Moderate	India	1986-2006	Cross-sectional	Multi-center	Unclear/not described	Other hospital based (not antenatal care)	Retrospective	Consecutive	25.6	1.20%	4.80%	NR	NR	NR
Bista	**2006**	19	Jaundice	High	Nepal	1999-2003	Cross-sectional	Single-center	Urban	Other hospital based (not antenatal care)	Retrospective	Unclear/not described	24	NR	NR	NR	NR	62.5
Borkakoti	**2013**	585	AVH and ALF	Moderate	India	2006-2011	Cross-sectional	Single-center	Urban	Other hospital based (not antenatal care)	Prospective	Unclear/not described	24.6	1.40%	2.40%	8.20%	NR	NR
Brohi	**2013**	52	FHF	Moderate	Pakistan	2009-2010	Cross-sectional	Single-center	Urban, Rural	Other hospital based (not antenatal care)	Prospective	Unclear/not described	29.04	NR	17.30%	13.50%	NR	NR
Cevrioglu	**2004**	245	None	Moderate	Turkey	2000-2002	Cross-sectional	Multi-center	Urban, Rural	Other hospital based (not antenatal care)	Prospective	Unclear/not described	26.3	NR	NR	NR	NR	NR
Changede	**2018**	43	Jaundice	Moderate	India	2016	Cross-sectional	Single-center	Urban, Rural	Other hospital based (not antenatal care)	Retrospective	Unclear/not described	NR	2.30%	4.60%	NR	NR	51
Cong	**2015**	990	None	Moderate	China	2011-2013	Cross-sectional	Multi-center	Urban, Rural	Antenatal care, Other hospital based (not antenatal care)	Prospective	Consecutive	NR	NR	NR	NR	NR	NR
Cordova	**2007**	20	None	Moderate	Brazil	2004	Cross-sectional	Multi-center	Urban	Other hospital based (not antenatal care)	Prospective	Unclear/not described	NR	NR	NR	NR	NR	NR
De Paschale	**2016**	278	None	Moderate	Benin	2011	Cross-sectional	Single-center	Rural	Other hospital based (not antenatal care)	Retrospective	Unclear/not described	26.2	0	NR	NR	NR	19
Devi	**2014**	262	AVH	Moderate	India	NR	Cross-sectional	Multi-center	Unclear/not described	Antenatal care, Other hospital based (not antenatal care)	Prospective	Unclear/not described	NR	NR	NR	NR	NR	NR
Elduma	**2014**	39	Fever	Moderate	Sudan	2012	Cross-sectional	Single-center	Urban	Other hospital based (not antenatal care)	Prospective	Unclear/not described	26	NR	NR	NR	NR	NR
Farshadpour	**2018**	1331	None	Low	Iran	2016-2017	Cross-sectional	Multi-center	Urban	Other hospital based (not antenatal care), Population/Community-based study	Prospective	Consecutive	27.93	NR	NR	NR	NR	5.1
Hamid	**1996**	12	AVH	Moderate	Pakistan	1991-1994	Cross-sectional	Unclear/not described	Urban	Unclear/not described	Prospective	Unclear/not described	25	8.30%	16.70%	0	NR	25
Hannachi	**2011**	404	None	Low	Tunisia	2006	Cross-sectional	Single-center	Urban, Rural	Antenatal care	Prospective	Consecutive	30.08	10%	0.03	0.50%	NR	NR

Huang	**2015**	274	None	High	China	2015	Cross-sectional	Single-center	Urban	Population/Community-based study	Prospective	Unclear/not described	NR	NR	NR	NR	NR	NR
Huang	**2013**	293	None	Moderate	China	NR	Cross-sectional	Single-center	Urban	Unclear/not described	Prospective	Unclear/not described	27.9	NR	NR	NR	NR	NR
Jaiswal	**2001**	127	AVH and FHF	Moderate	India	1992_1999	Cross-sectional	Single-center	Urban	Other hospital based (not antenatal care)	Prospective	Consecutive	NR	0	18.89%	0	0.78%	NR
Javed	**2017**	135	Jaundice or abnormal liver function	Moderate	Pakistan	2015	Cross-sectional	Multi-center	Urban	Other hospital based (not antenatal care)	Prospective	Unclear/not described	29.2	NR	5.20%	5.20%	NR	15
Jilani	**2007**	50	FHF	Moderate	India	NR	Cross-sectional	Multi-center	Unclear/not described	Other hospital based (not antenatal care)	Prospective	Unclear/not described	24.8	0.02	0	0	NR	NR
Junaid	**2014**	108	None	Moderate	Nigeria	NR	Cross-sectional	Single-center	Urban, Rural	Population/Community-based study	Prospective	Non-probability	NR	NR	NR	NR	NR	NR
Kar	**2008**	125	Jaundice	Low	India	NR	Cross-sectional	Multi-center	Urban	Antenatal care, Other hospital based (not antenatal care)	Prospective	Consecutive	24.8	12%	0.08	0.02	NR	NR
Khuroo	**2003**	49	ALF	Low	India	1989-1996	Cross-sectional	Single-center	Urban	Other hospital based (not antenatal care)	Prospective	Consecutive	31.1	2.20%	13.90%	7.20%	1.10%	NR
Khuroo	**2003**	76	AVH	Low	India	1993-1996	Cross-sectional	Single-center	Urban	Other hospital based (not antenatal care)	Prospective	Systematic	27.7	1.50%	0.15	1.70%	1.50%	NR
Kumar	**2004**	62	Jaundice	Moderate	India	2001-2002	Cross-sectional	Unclear/not described	Unclear/not described	Antenatal care, Other hospital based (not antenatal care)	Prospective	Consecutive	24.5	0	4.80%	0	NR	NR
Kumar	**2011**	24	None	Moderate	India	2002-2006	Cross-sectional	Single-center	Urban	Unclear/not described	Retrospective	Unclear/not described	NR	0	8.30%	0	NR	NR
Kumar	**2017**	256	Jaundice	Moderate	India	2011-2014	Cross-sectional	Multi-center	Urban	Antenatal care, Other hospital based (not antenatal care)	Prospective	Consecutive	24.6	0.07%	3.40%	3.40%	NR	0
Kumar	**2007**	24	Jaundice	High	India	2002-2006	Cross-sectional	Single-center	Urban	Other hospital based (not antenatal care)	Prospective	Unclear/not described	NR	14.60%	12.20%	3.60%	NR	NR
Kumar	**2017**	177	Jaundice	Moderate	India	2016	Cross-sectional	Single-center	Urban	Other hospital based (not antenatal care)	Retrospective	Unclear/not described	25	0.40%	30.50%	8.40%	NR	NR
Kumar	**2001**	469	None	Low	United Arab Emirates	NR	Cross-sectional	Single-center	Urban	Antenatal care	Prospective	Consecutive	25	NR	NR	NR	NR	NR
Lindemann	**2010**	1040	None	Moderate	Spain	2007-2010	Cross-sectional	Single-center	Urban	Other hospital based (not antenatal care)	Prospective	Unclear/not described	30.6	NR	NR	NR	NR	NR
Mahtab	**2009**	31	AVH	Moderate	Bangladesh	2004-2006	Cross-sectional	Single-center	Urban	Other hospital based (not antenatal care)	Retrospective	Consecutive	NR	4.86%	27.80%	NR	NR	NR
Neffati	**2017**	216	None	Low	Tunisia	2014-2015	Cross-sectional	Multi-center	Urban	Antenatal care	Prospective	Consecutive	32	9.80%	NR	NR	NR	NR
Obiri-Yeboah	**2018**	398	None	Moderate	Ghana	NR	Cross-sectional	Multi-center	Urban, Rural	Antenatal care	Prospective	Systematic	28.01	NR	NR	NR	NR	NR
Oncu	**2006**	386	None	Moderate	Turkey	NR	Cross-sectional	Multi-center	Urban, Rural	Other hospital based (not antenatal care)	Prospective	Multistage sampling	24.28	NR	NR	NR	NR	NR
Patra	**2007**	220	Jaundice	Low	India	2003-2005	Cross-sectional	Multi-center	Urban	Other hospital based (not antenatal care)	Prospective	Consecutive	22.4	NR	NR	NR	NR	NR
Pujol	**1994**	184	None	Moderate	Venezuela	1991-1992	Cross-sectional	Multi-center	Urban, Rural	Other hospital based (not antenatal care)	Prospective	Unclear/not described	NR	NR	NR	NR	NR	NR

Rathi	**2007**	107	Liver dysfunction	Moderate	India	2002-2004	Cross-sectional	Single-center	Urban	Other hospital based (not antenatal care)	Prospective	Consecutive	NR	NR	4.20%	4.20%	NR	NR
Renou	**2014**	315	None	Moderate	France	NR	Cross-sectional	Multi-center	Unclear/not described	Other hospital based (not antenatal care)	Prospective	Unclear/not described	NR	NR	NR	NR	NR	NR
Sahai	**2015**	68	AVL	Moderate	India	2007-2009	Cross-sectional	Unclear/not described	Unclear/not described	Unclear/not described	Prospective	Consecutive	NR	0	7.40%	1.50%	NR	NR
Salam	**2013**	272	Jaundice	High	India	2008-2011	Cross-sectional	Multi-center	Unclear/not described	Antenatal care	Prospective	Unclear/not described	24.76	1.80%	8.80%	2.70%	NR	NR
Singh	**2003**	60	AVH	Moderate	India	1997-1998	Cross-sectional	Multi-center	Urban	Other hospital based (not antenatal care)	Prospective	Unclear/not described	24	0	0.1	0	NR	NR
Solanke	**2016**	103	Liver dysfunction	Moderate	India	2013-2015	Cross-sectional	Unclear/not described	Urban	Other hospital based (not antenatal care)	Prospective	Consecutive	25	NR	NR	NR	NR	NR
Strand	**2003**	60	Both	Moderate	Angola	1999	Case-control (group of pregnant women)	Multi-center	Urban	Other hospital based (not antenatal care)	Prospective	Consecutive	21.9	NR	0.1	0	NR	55
Sultana	**2014**	38	Jaundice	Moderate	Pakistan	2012-2013	Cross-sectional	Single-center	Urban	Other hospital based (not antenatal care)	Prospective	Unclear/not described	25	12%	NR	NR	NR	40
Surya	**2005**	819	None	Moderate	Indonesia	2003	Cross-sectional	Multi-center	Urban	Other hospital based (not antenatal care)	Prospective	Unclear/not described	27	NR	1.90%	0.04%	NR	NR

NR: not reported; AVH: acute viral hepatitis, FHF: fulminant hepatitis failure; ALF: acute liver failure

Supplementary Table 5 : ***Individual characteristics of studies included in the meta-analysis of pregnancy outcomes in HEV infection in the global population of pregnant women***

Author	Year of publication	Country	Design	Number of centers	Area	Setting	Timing of data collection	Period	Sampling	Age HEV+	Age HEV-	%HAV in HEV+	%HAV in HEV-	%HBV in HEV+	%HBV in HEV-	%HCV in HEV+	%HCV in HEV-	%HDV in HEV+	%HDV in HEV-	%HIV in HEV+	%HIV in HEV-	%Nulliparous in HEV+	%Nulliparous in HEV-
**Patra**	2007	India	Cross-sectional	Multi-center	Urban	Other hospital based (not antenatal care)	Prospective	2003-2005	Consecutive	NR	NR	NR	NR	NR	NR	NR	NR	NR	NR	NR	NR	NR	NR
**Kumar**	2014	India	Cross-sectional	Multi-center	Unclear/not described	Antenatal care, Other hospital based (not antenatal care)	Unclear/not described	NR	Unclear/not described	NR	NR	NR	NR	NR	NR	NR	NR	NR	NR	NR	NR	NR	NR
**Devi**	2014	India	Cross-sectional	Multi-center	Unclear/not described	Antenatal care, Other hospital based (not antenatal care)	Unclear/not described	NR	Unclear/not described	NR	NR	NR	NR	NR	NR	NR	NR	NR	NR	NR	NR	NR	NR
**Bhatnagar**	2016	India	Cross-sectional	Multi-center	Urban	Antenatal care, Other hospital based (not antenatal care)	Prospective	2012-2014	Consecutive	24.10	25.57	NR	NR	NR	NR	NR	NR	NR	NR	NR	NR	NR	NR
**Javed**	2017	Pakistan	Cross-sectional	Multi-center	Unclear/not described	Unclear/not described	Prospective	2015	Unclear/not described	29.2	28.6	NR	NR	2.2%	3.0%	0.7%	4.4%	NR	NR	NR	NR	NR	NR

NR: Not reported

Supplementary Table 6 : ***Characteristics of studies included in the meta-analysis***

**Characteristics**	**N = 54**
Year or publication, range	1994-2018
Period of recruitment, range	1986-2017 (n = 42)
% of Nulliparous	0 – 62.5 (n = 11)
Clinical profile	
- Asymptomatic	24
- Symptomatic	29
- Both	1
WHO Regions	
- South-East Asia	25
- Eastern Mediterranean	10
- Africa	7
- Europe	4
- Americas	3
- Western Pacific	3
Design	
- Cross sectional	53
- Case control	1
Setting	
- Antenatal care	14
- Hospital-based	34
- Population-based	2
- Unclear	4
Timing of data collection	
- Prospective	45
- Retrospective	9
Sampling method	
- Consecutive	25
- Multistage random	1
- Random sampling	1
- Systematic	2
- Unclear	25

Supplementary Figure 1 : ***The review process***

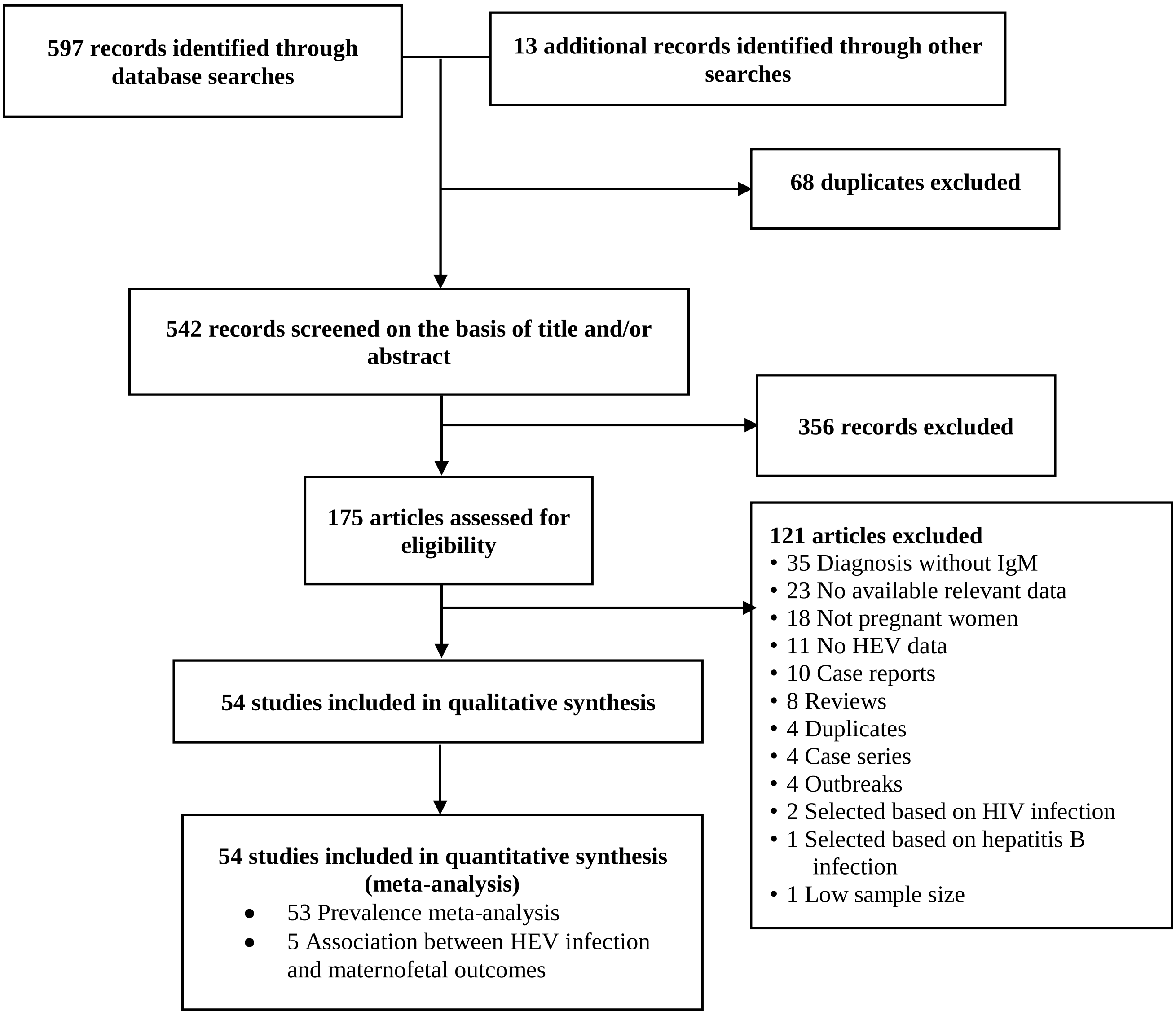
597 records identified through database searches
13 additional records identified through other searches
68 duplicates excluded
542 records screened on the basis of title and/or abstract
356 records excluded
175 articles assessed for eligibility
121 articles excluded
• 35 Diagnosis without IgM
• 23 No available relevant data
• 18 Not pregnant women
• 11 No HEV data
• 10 Case reports
• 8 Reviews
• 4 Duplicates
• 4 Case series
• 4 Outbreaks
• 2 Selected based on HIV infection
• 1 Selected based on hepatitis B infection
• 1 Low sample size
54 studies included in qualitative synthesis
54 studies included in quantitative synthesis (meta-analysis)
• 53 Prevalence meta-analysis
• 5 Association between HEV infection and maternofetal outcomes

Supplementary Figure 2 : ***Funnel plot for HEV prevalence in asymptomatic pregnant women***

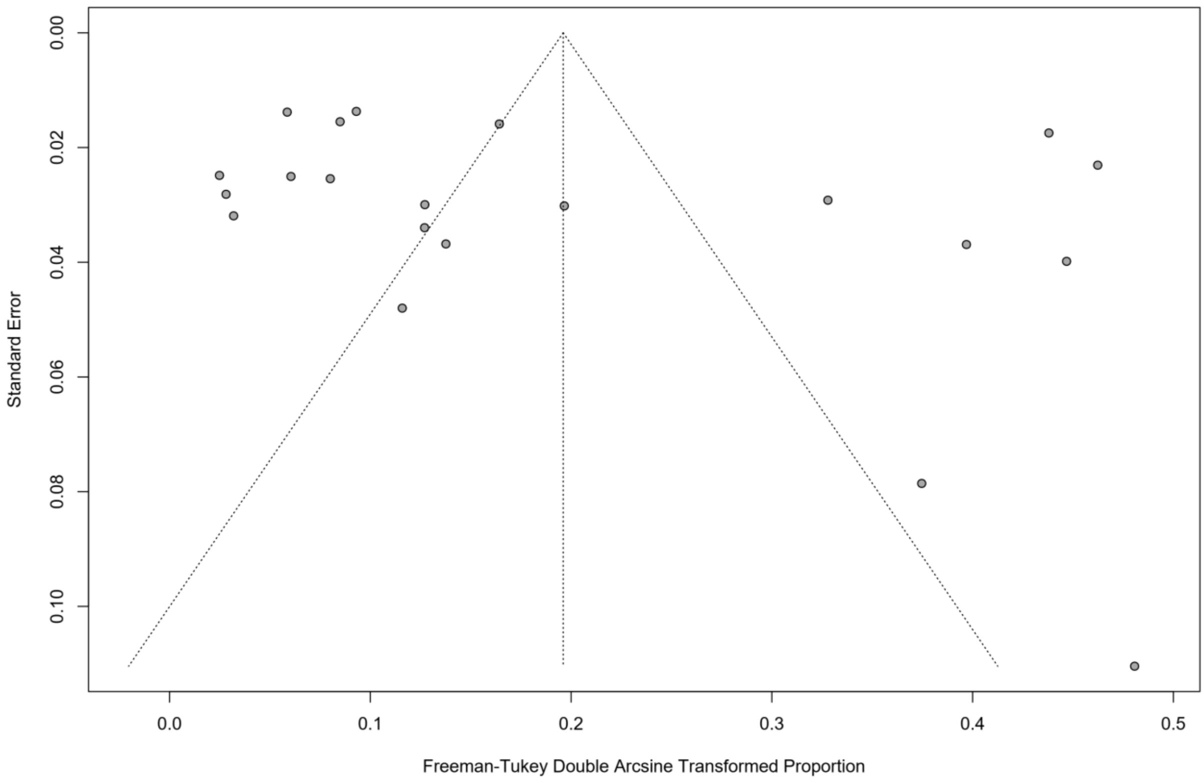

Supplementary Figure 3 : ***Funnel plot for HEV prevalence in symptomatic pregnant women***

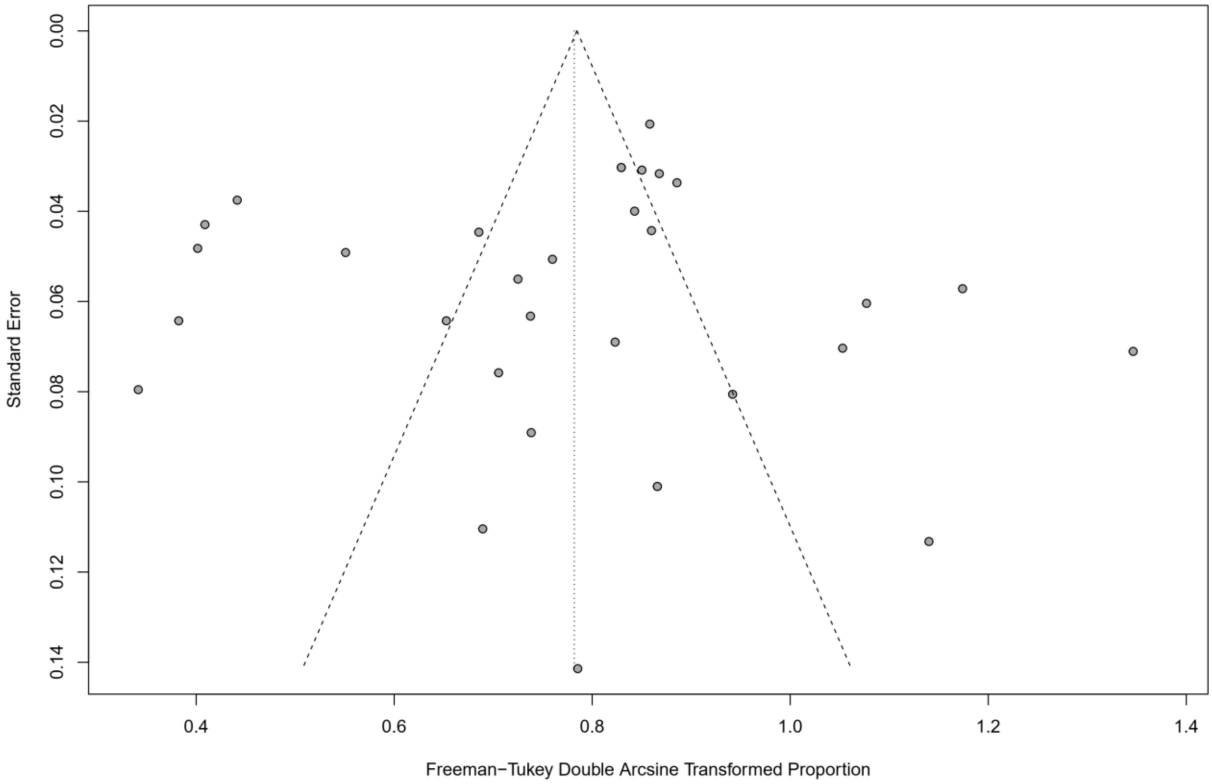

List of included studies[1-54]

[1] Bhatnagar G, Sharma S, Kumar A, Prasad S, Agarwal S, Kar P. Reduced glutathione in hepatitis E infection and pregnancy outcome. Journal of Obstetrics and Gynaecology Research 2016;42:789-795.

[2] Devi SG, Kumar A, Kar P, Husain SA, Sharma S. Association of pregnancy outcome with cytokine gene polymorphisms in HEV infection during pregnancy. Journal of medical virology 2014;86:1366-1376.

[3] Javed NA, Ullah SH, Hussain N, Sheikh MA, Khan A, Ghafoor F, et al. Hepatitis e virus seroprevalence in pregnant women in Pakistan: Maternal and fetal outcomes. Eastern Mediterranean Health Journal 2017;23:559-563.

[4] Kumar A, Devi SG, Kar P, Agarwal S, Husain SA, Gupta RK, et al. Association of cytokines in hepatitis E with pregnancy outcome. Cytokine 2014;65:95-104.

[5] Patra S, Kumar A, Trivedi SS, Puri M, Sarin SK. Maternal and fetal outcomes in pregnant women with acute hepatitis E virus infection. Annals of Internal Medicine 2007;147:28-33.

[6] Abebe M, Ali I, Ayele S, Overbo J, Aseffa A, Mihret A. Seroprevalence and risk factors of Hepatitis E Virus infection among pregnant women in Addis Ababa, Ethiopia. PLoS ONE 2017;12.

[7] Adjei AA, Tettey Y, Aviyase JT, Adu-Gyamfi C, Obed S, Mingle JA, et al. Hepatitis e virus infection is highly prevalent among pregnant women in Accra, Ghana. Virology Journal 2009;6.

[8] Ahmed RE, Karsany MS, Adam I. Brief report: Acute viral hepatitis and poor maternal and perinatal outcomes in pregnant Sudanese women. Journal of medical virology 2008;80:1747-1748.

[9] Singla A, Mehta S, Rajaram S, Shree S. Materno-Fetal Outcomes with Viral Hepatitis in Pregnancy. Journal of Obstetrics and Gynecology of India 2016;66:166-169.

[10] Banait VS, Sandur V, Parikh F, Murugesh M, Ranka P, Ramesh VS, et al. Outcome of acute liver failuredue to acute hepatitis E in pregnant women. Indian Journal of Gastroenterology 2007;26:6-10.

[11] Beniwal M, Kumar A, Kar P, Jilani N, Sharma JB. Prevalence and severity of acute viral hepatitis and fulminant hepatitis during pregnancy: A prospective study from North India. Indian Journal of Medical Microbiology 2003;21:184-185.

[12] Bhatia V, Singhal A, Panda SK, Acharya SK. A 20-year single-center experience with acute liver failure during pregnancy: Is the prognosis really worse? Hepatology (Baltimore, Md) 2008;48:1577-1585.

[13] Bista BK, Rana A. Acute hepatitis E in pregnancy--study of 16 cases. JNMA; journal of the Nepal Medical Association 2006;45:182-185.

[14] Borkakoti J, Hazam RK, Mohammad A, Kumar A, Kar P. Does high viral load of hepatitis E virus influence the severity and prognosis of acute liver failure during pregnancy? Journal of medical virology 2013;85:620-626.

[15] Brohi ZP, Sadaf A, Perveen U. Etiology, clinical features and outcome of fulminant hepatic failure in pregnancy. Journal of the Pakistan Medical Association 2013;63:1168-1171.

[16] Cordova CMMd, Blatt SL, Botelho TKR, Dalmarco EM. Sorologia para o vírus da Hepatite E em gestantes: clinicamente importante ou desnecessário? Rev bras anal clin 2007;39:269-273.

[17] Cevrioglu AS, Altindis M, Tanir HM, Aksoy F. Investigation of the incidence of hepatitis E virus among pregnant women in Turkey. Journal of Obstetrics and Gynaecology Research 2004;30:48-52.

[18] Changede P, Chavan N, Raj N, Gupta P. An Observational Study to Evaluate the Maternal and Foetal Outcomes in Pregnancies Complicated with Jaundice. Journal of Obstetrics and Gynecology of India 2018:1-6.

[19] Cong W, Sui JC, Zhang XY, Qian AD, Chen J, Zhu XQ. Seroprevalence of hepatitis E virus among pregnant women and control subjects in China. Journal of medical virology 2015;87:446-450.

[20] De Paschale M, Ceriani C, Romanò L, Cerulli T, Cagnin D, Cavallari S, et al. Epidemiology of hepatitis E virus infection during pregnancy in Benin. Tropical Medicine and International Health 2016;21:108-113.

[21] Elduma AH, Osman WM. Dengue and hepatitis E virus infection in pregnant women in Eastern Sudan, A challenge for diagnosis in an endemic area. Pan African Medical Journal 2014;19.

[22] Farshadpour F, Taherkhani R, Ravanbod MR, Eghbali SS, Taherkhani S, Mahdavi E. Prevalence, risk factors and molecular evaluation of hepatitis E virus infection among pregnant women resident in the northern shores of Persian Gulf, Iran. PLoS ONE 2018;13.

[23] Hamid SS, Jafri SMW, Khan H, Shah H, Abbas Z, Fields H. Fulminant hepatic failure in pregnant women: Acute fatty liver or acute vital hepatitis? Journal of Hepatology 1996;25:20-27.

[24] Hannachi N, Hidar S, Harrabi I, Mhalla S, Marzouk M, Ghzel H, et al. Seroprevalence and risk factors of hepatitis E among pregnant women in central Tunisia. Pathologie Biologie 2011;59:e115-e118.

[25] Huang F, Ma T, Li L, Zeng W, Jing S. Low seroprevalence of hepatitis E virus infection in pregnant women in Yunnan, China. The Brazilian journal of infectious diseases : an official publication of the Brazilian Society of Infectious Diseases 2013;17:716-717.

[26] Huang F, Wang J, Yang C, Long F, Li Y, Li L, et al. Chinese pregnant women in their third trimester are more susceptible to HEV infection. The Brazilian journal of infectious diseases : an official publication of the Brazilian Society of Infectious Diseases 2015;19:672-674.

[27] Jaiswal SPB, Jain AK, Naik G, Soni N, Chitnis DS. Viral hepatitis during pregnancy. International Journal of Gynecology and Obstetrics 2001;72:103-108.

[28] Jilani N, Das BC, Husain SA, Baweja UK, Chattopadhya D, Gupta RK, et al. Hepatitis E virus infection and fulminant hepatic failure during pregnancy. Journal of Gastroenterology and Hepatology (Australia) 2007;22:676-682.

[29] Junaid SA, Agina SE, Abubakar KA. Epidemiology and associated risk factors of hepatitis E virus infection in Plateau State, Nigeria. Virology: Research and Treatment 2014;5:15-26.

[30] Kar P, Jilani N, Husain SA, Pasha ST, Anand R, Rai A, et al. Does hepatitis E viral load and genotypes influence the final outcome of acute liver failure during pregnancy? American Journal of Gastroenterology 2008;103:2495-2501.

[31] Khuroo MS, Kamili S. Aetiology, clinical course and outcome of sporadic acute viral hepatitis in pregnancy. Journal of Viral Hepatitis 2003;10:61-69.

[32] Khuroo MS, Kamili S. Aetiology and prognostic factors in acute liver failure in India. Journal of Viral Hepatitis 2003;10:224-231.

[33] Kumar A, Sharma S, Kar P, Agarwal S, Ramji S, Husain SA, et al. Impact of maternal nutrition in hepatitis E infection in pregnancy. Archives of Gynecology and Obstetrics 2017;296:885-895.

[34] Kumar S, Pujhari SK, Chawla YK, Chakraborti A, Ratho RK. Molecular detection and sequence analysis of hepatitis E virus in patients with viral hepatitis from North India. Diagnostic Microbiology and Infectious Disease 2011;71:110-117.

[35] Kumar RM, Uduman S, Rana S, Kochiyil JK, Usmani A, Thomas L. Sero-prevalence and mother-to-infant transmission of hepatitis E virus among pregnant women in the United Arab Emirates. European Journal of Obstetrics Gynecology and Reproductive Biology 2001;100:9-15.

[36] Kumar N, Das V, Agarwal A, Pandey A, Agrawal S. Fetomaternal outcomes in pregnant women with hepatitis e infection; still an important fetomaternal killer with an unresolved mystery of increased virulence in pregnancy. Turk Jinekoloji ve Obstetrik Dernegi Dergisi 2017;14:106-113.

[37] Kumar S, Ratho RK, Chawla YK, Chakraborti A. The incidence of sporadic viral hepatitis in North India: A preliminary study. Hepatobiliary and Pancreatic Diseases International 2007;6:596-599.

[38] Kumar A, Beniwal M, Kar P, Sharma JB, Murthy NS. Hepatitis E in pregnancy. International Journal of Gynecology and Obstetrics 2004;85:240-244.

[39] Lindemann MLM, Gabilondo G, Romero B, De La Maza OMS, Pérez-Gracia MT. Low prevalence of hepatitis E infection among pregnant women in Madrid, Spain. Journal of medical virology 2010;82:1666-1668.

[40] Mahtab MA, Rahman S, Khan M, Mamum AA, Afroz S. Etiology of fulminant hepatic failure: Experience from a tertiary hospital in Bangladesh. Hepatobiliary and Pancreatic Diseases International 2008;7:161-164.

[41] Neffatti H, Lebraud P, Hottelet C, Gharbi J, Challouf T, Roque-Afonso AM. Southern Tunisia: A still high endemicity area for hepatitis A. PLoS ONE 2017;12.

[42] Obiri-Yeboah D, Awuku YA, Adu J, Pappoe F, Obboh E, Nsiah P, et al. Sero-prevalence and risk factors for hepatitis E virus infection among pregnant women in the Cape Coast Metropolis, Ghana. PLoS ONE 2018;13.

[43] Pujol FH, Favorov MO, Marcano T, Este JA, Magris M, Liprandi F, et al. Prevalence of antibodies against hepatitis E virus among urban and rural populations in Venezuela. Journal of medical virology 1994;42:234-236.

[44] Rathi U, Bapat M, Rathi P, Abraham P. Effect of liver disease on maternal and fetal outcome a prospective study. Indian Journal of Gastroenterology 2007;26:59-63.

[45] Renou C, Gobert V, Locher C, Moumen A, Timbely O, Savary J, et al. Prospective study of Hepatitis e Virus infection among pregnant women in France. Virology Journal 2014;11.

[46] Sahai S, Mishra V, Ganga D, Jatav OP. Viral Hepatitis in Pregnancy--A study of its Effect on Maternal and Foetal Outcome. The Journal of the Association of Physicians of India 2015;63:28-33.

[47] Salam GD, Kumar A, Kar P, Aggarwal S, Husain A, Sharma S. Serum tumor necrosis factor-alpha level in hepatitis E virus-related acute viral hepatitis and fulminant hepatic failure in pregnant women. Hepatology Research 2013;43:826-835.

[48] Singh S, Mohanty A, Joshi YK, Deka D, Mohanty S, Panda SK. Mother-to-child transmission of hepatitis E virus infection. Indian Journal of Pediatrics 2003;70:37-39.

[49] Solanke D, Rathi C, Pandey V, Patil M, Phadke A, Sawant P. Etiology, clinical profile, and outcome of liver disease in pregnancy with predictors of maternal mortality: A prospective study from Western India. Indian Journal of Gastroenterology 2016;35:450-458.

[50] Strand RT, Franque-Ranque M, Bergström S, Weiland O. Infectious aetiology of jaundice among pregnant women in Angola. Scandinavian Journal of Infectious Diseases 2003;35:401-403.

[51] Sultana R, Humayun S. Fetomaternal outcome in acute hepatitis E. Journal of the College of Physicians and Surgeons Pakistan 2014;24:127-130.

[52] Surya IGP, Kornia K, Suwardewa TGA, Mulyanto, Tsuda F, Mishiro S. Serological markers of hepatitis B, C, and E viruses and human immunodeficiency virus type-1 infections in pregnant women in Bali, Indonesia. Journal of medical virology 2005;75:499-503.

[53] Tejada-Strop A, Tohme RA, Andre-Alboth J, Childs L, Ji X, De Oliveira Landgraf De Castro V, et al. Seroprevalence of Hepatitis A and Hepatitis e viruses among pregnant women in Haiti. American Journal of Tropical Medicine and Hygiene 2019;101:230-232.

[54] Modiyinji AF, Amougou-Atsama M, Monamele CG, Nola M, Njouom R. Seroprevalence of hepatitis E virus antibodies in different human populations of Cameroon. Journal of Medical Virology 2019;91:1989-1994.
